# Mesenchymal Stromal Cells Combined With Elastin-Like Recombinamers Increase Angiogenesis *In Vivo* After Hindlimb Ischemia

**DOI:** 10.3389/fbioe.2022.918602

**Published:** 2022-06-23

**Authors:** Arturo Ibáñez-Fonseca, Ana Rico, Silvia Preciado, Fernando González-Pérez, Sandra Muntión, Jesús García-Briñón, María-Carmen García-Macías, José Carlos Rodríguez-Cabello, Miguel Pericacho, Matilde Alonso, Fermín Sánchez-Guijo

**Affiliations:** ^1^ BIOFORGE Lab, University of Valladolid, CIBER-BBN, Valladolid, Spain; ^2^ Cell Therapy Unit, Hematology Department, University Hospital of Salamanca, Salamanca, Spain; ^3^ RICORS TERAV, ISCIII, Madrid, Spain; ^4^ Centro en Red de Medicina Regenerativa y Terapia Celular de Castilla y León, Salamanca, Spain; ^5^ Instituto de Investigación Biomédica de Salamanca (IBSAL), Salamanca, Spain; ^6^ Department of Medicine and Cancer Research Center, University of Salamanca, Salamanca, Spain; ^7^ Departamento de Biología Celular y Patología, Facultad de Medicina, Salamanca, Spain; ^8^ Renal and Cardiovascular Research Unit, Department of Physiology and Pharmacology, University of Salamanca, Salamanca, Spain

**Keywords:** mesenchymal stromal cells, biomaterials, elastin-like recombinamers, hindlimb ischemia, angiogenesis, mesenchymal stem cells

## Abstract

Hindlimb ischemia is an unmet medical need, especially for those patients unable to undergo vascular surgery. Cellular therapy, mainly through mesenchymal stromal cell (MSC) administration, may be a potentially attractive approach in this setting. In the current work, we aimed to assess the potential of the combination of MSCs with a proangiogenic elastin-like recombinamer (ELR)–based hydrogel in a hindlimb ischemia murine model. Human bone marrow MSCs were isolated from four healthy donors, while ELR biomaterials were genetically engineered. Hindlimb ischemia was induced through ligation of the right femoral artery, and mice were intramuscularly injected with ELR biomaterial, 0.5 × 10^6^ MSCs or the combination, and also compared to untreated animals. Tissue perfusion was monitored using laser Doppler perfusion imaging. Histological analysis of hindlimbs was performed after hematoxylin and eosin staining. Immunofluorescence with anti–human mitochondria antibody was used for human MSC detection, and the biomaterial was detected by elastin staining. To analyze the capillary density, immunostaining with an anti–CD31 antibody was performed. Our results show that the injection of MSCs significantly improves tissue reperfusion from day 7 (*p* = 0.0044) to day 21 (*p* = 0.0216), similar to the infusion of MSC + ELR (*p* = 0.0038, *p* = 0.0014), without significant differences between both groups. After histological evaluation, ELR hydrogels induced minimal inflammation in the injection sites, showing biocompatibility. MSCs persisted with the biomaterial after 21 days, both *in vitro* and *in vivo*. Finally, we observed a higher blood vessel density when mice were treated with MSCs compared to control (p<0.0001), but this effect was maximized and significantly different to the remaining experimental conditions when mice were treated with the combination of MSCs and the ELR biomaterial (*p* < 0.0001). In summary, the combination of an ELR-based hydrogel with MSCs may improve the angiogenic effects of both strategies on revascularization of ischemic tissues.

## Introduction

Hindlimb ischemia is the final stage of peripheral vascular disease and is of great prevalence in developed countries, with enormous morbidity and mortality rates (Sontheimer, 2006). It has an ineffective treatment in cases where vascular surgery has failed or cannot be performed (Sontheimer, 2006). Therefore, it is vital to find a potentially effective strategy in this context that may promote angiogenesis, which is a crucial process for ensuring tissue maintenance and functionality. Angiogenesis is the formation of new blood vessels from pre-existing ones, and abnormalities in this process may lead to blood flow deprivation, apoptosis, necrosis, inflammation, and decreased capillary density (Cooke and Meng, 2020). Two potential therapeutic options to revert ischemic damage can be the delivery of pro-angiogenic factors and use of progenitor cells (Pacilli et al., 2010). Regarding the latter, diverse evidence supports that a cell-based therapy may provide promising outcomes in the treatment of ischemia (Ahmad et al., 2007; Cao et al., 2009; Sanchez-Guijo et al., 2010; Sun et al., 2013).

Bone marrow–derived mesenchymal stromal cells (BM-MSCs) are known for their therapeutic potential due to their multilineage differentiation potential and their immunomodulatory and anti-inflammatory capacity (Valencia et al., 2016), and they are increasingly being used in cell therapy and regenerative medicine programs (Zhang et al., 2013; Xiao et al., 2015; Cai et al., 2016). MSCs are involved in the regenerating process of the damaged tissues through different mechanisms, including the release of bioactive molecules either directly or through extracellular vesicles (EVs), which exert effects on the surrounding cells and their capacity to differentiate into different types of functional cells (Barry and Murphy, 2004; Timmers et al., 2008). Previous experimental and clinical data have demonstrated the therapeutic effects of BM-derived stem cells injected in the muscle of mice with hindlimb ischemic injury (Kinnaird et al., 2004; Lian et al., 2010; Hu et al., 2015). BM-MSCs enhance blood flow recovery and capillary density through neo-vascularization with capillaries and small vessels. However, for its adequate therapeutic use in some applications, MSCs need to be administered together with biomaterials that act as carriers, allowing their adequate implantation and retention and favoring the functionality of the cells (Roche et al., 2014; Jeong et al., 2018; García-Sánchez et al., 2019; Zhao et al., 2021).

Different studies report that biomaterial scaffolds alone may stimulate cell growth, differentiation, and promote angiogenesis (Serbo and Gerecht, 2013; Mehdizadeh et al., 2015). Furthermore, biomaterials can provide mechanical support and induce regeneration in the damaged tissues. However, the selection of the scaffold is crucial in ensuring its successful engraftment to promote tissue regeneration. In this regard, Mehdizadeh et al. showed how angiogenesis and host integration of the engineered tissues can easily be controlled by modulating the functionalization of the biomaterial alone.

The biomaterials used in this study are based on the structure of natural elastin, comprising its main features, such as thermoresponsiveness and elasticity. Due to their recombinant origin, they have been termed elastin-like recombinamers (ELRs) (González de Torre et al., 2014), and their protein backbone mainly comprises repetitions of the L-Val–L-Pro–Gly–X-Gly (VPGXG) pentapeptide, in which X (guest residue) can be any amino acid except for L-Pro. Therefore, the inclusion of amino acids with highly reactive groups in their side chain permits the modification of the ELRs for specific purposes. For instance, the use of lysine residues allows the conversion of their -amine groups to bear azide or cyclooctyne groups, which react bioorthogonally through “click-chemistry” when mixed in solution, forming a molecular network that eventually yields a covalently cross-linked hydrogel in a short time (González de Torre et al., 2014). Moreover, the recombinant technology enables the inclusion of other bioactive peptide sequences to improve the functionality of the ELRs.

In the present work, we have chosen two previously designed ELRs that are able to form chemical hydrogels and that incorporate two different types of cell adhesion domains, both of them found in fibronectin: the L-Arg–Gly–L-Asp (RGD) tripeptide, which promotes good adhesion of almost every cell type mainly *via* α_v_β_3_ and α_5_β_1_ integrins (Ruoslahti and Pierschbacher, 1986; Ruoslahti, 1996), and the L-Arg–L-Glu–L-Asp–L-Val (REDV) tetrapeptide that induces the specific adhesion of endothelial cells through adhesion to α_4_β_1_ integrins (Massia and Hubbell, 1992; Girotti et al., 2004). In addition, this latter recombinamer also includes L-Val–Gly–L-Val–L-Ala–L-Pro–Gly (VGVAPG) elastase (also known as MMP-12)–sensitive motifs, facilitating its enzymatic biodegradation (Taddese et al., 2009). Thus, we can obtain pro-angiogenic (biodegradable and bioactive) hydrogels with these ELRs, which are also injectable due to the control of the gelation time upon mixing both ELR-azide and ELR-cyclooctyne solutions. Hydrogels based on ELRs are being increasingly used because of their biocompatibility (Ibáñez-Fonseca et al., 2018), and they have already proven their ability to promote tissue regeneration (e.g., osteochondral, bone, skin, skeletal muscle tissue, and myocardium) (Coletta et al., 2017; Pescador et al., 2017; Ibáñez-Fonseca et al., 2020; Contessotto et al., 2021; Stojic et al., 2021). Moreover, Staubli et al. demonstrated that the use of the aforementioned ELR-based hydrogels promotes cell adhesion, *in vivo* angiogenesis, and integration into the host tissue (Staubli et al., 2017). All these properties allow this class of ELR-based hydrogels to be a versatile cell-delivery platform for diverse tissue engineering strategies, including ischemia regeneration.

In this study, our purpose is to analyze the possible contribution of BM-MSCs with or without a pro-angiogenic hydrogel to angiogenesis in a hindlimb ischemia murine model, analyzing blood flow and capillary density in the hindlimbs. This study can set the preclinical basis for a subsequent clinical trial with BM-MSCs and ELR-based hydrogels that assess the safety and efficacy of this therapeutic approach.

## Materials and Methods

### Bone Marrow Mesenchymal Stem Cells

Human BM-MSCs were isolated from four healthy donors (two men and two women) with a median age of 37 years (range, 26–41). In all cases, written informed consent was previously obtained according to institutional guidelines and the Declaration of Helsinki. All experimental procedures were also approved by the Ethics Committee of the Hospital Universitario de Salamanca.

Ten to 20 ml BMs were obtained from the iliac crest under local anesthesia. BM mononuclear cells were isolated using density-gradient centrifugation with Ficoll–Paque (Ficoll–Paque density, 1.077 g/ml; GE Healthcare Bio Sciences, AB, Uppsala, Sweden). The cells were counted and seeded at a density of 1 × 10^6^ cells/cm^2^, and expansion was carried out as previously described (Carrancio et al., 2008) in DMEM (GIBCO, Life Technologies, Carlsbad, CA) supplemented with 10% FBS (GIBCO, Life Technologies) and 1% penicillin/streptomycin at 37°C in a humidified atmosphere with 5% of CO_2_. The culture medium was changed every 3–4 days. All the experiments were performed using cultured BM-MSCs at passages 3–6.

### Bioproduction, Characterization, and Chemical Modification of the Elastin-Like Recombinamers

The bioproduction of the ELRs has been extensively described before (Rodríguez-Cabello et al., 2012), and they were obtained from Technical Proteins Nanobiotechnology, S. L. (Spain). Briefly, they were genetically engineered to comprise the genes encoding for the elastin-like motifs, in combination with cell adhesion sequences, using the iterative recursive method, and finally cloned into a pET-25b(+) plasmid vector (Novagen, Merck KGaA, Germany), subsequently used to transform a BLR(DE3) strain of *E. coli* (Novagen, Merck KGaA, Germany). Then, bacteria were cultured in a 15-L bioreactor (Applikon Biotechnology B.V., Netherlands), and the recombinamers were purified through inverse temperature cycling combining heating and cooling steps followed by centrifugation, taking advantage of their thermoresponsiveness. Once purified, the ELRs were dialyzed against ultrapure water and sterile-filtered prior to freeze-drying. Through this procedure, two different ELRs were obtained: the so-called HRGD6 and REDV. The first comprises the Arg–Gly–Asp (RGD) motif, while the latter includes the Arg–Glu–Asp–Val (REDV) amino acid sequences. Both of them promote cell adhesion, with REDV being more specific toward endothelial cells. In addition, the REDV recombinamer also contains the Val–Gly–Val–Ala–Pro–Gly (VGVAPG) sequence, which is sensitive to elastase, hence conferring enzymatic biodegradation to the ELR.

The batch-to-batch characterization of the ELRs included SDS-PAGE for purity and molecular weight assessment, differential scanning calorimetry (DSC) for the determination of the transition temperature, and ^1^H-NMR to obtain an ELR fingerprint. All these and other methods were used in previous studies to fully characterize the ELRs used in this work (Girotti et al., 2004; Costa et al., 2011).

The ELRs were chemically modified as described elsewhere (González de Torre et al., 2014), through the transformation of the ε-amine group present in the lysine side chain to bear azide (N_3_) or cyclooctyne groups in order to achieve a “click-chemistry” reaction between ELR molecules to get a hydrogel network.

### ELR Hydrogel Formation

An HRGD6-Cyclo and a REDV-N_3_ were used for the formation of the hydrogels used in this work, combining broad (*via* RGD) and specific (*via* REDV for endothelial cells) cell adhesion and biodegradation by elastase-mediated cleavage. The hydrogels were formed through the combination of both ELRs in a 1:1 volume ratio at 50 mg/ml (dissolved in RPMI medium without serum or other additives), in every case. See the sections “*Cell and hydrogel administration*” and the “*3D culture*” for more details on the formation of hydrogels for these applications.

### Mouse Model and Animal Surgery

Animal care and procedures were conducted in compliance with Spanish and European Union guidelines (RD 1201/05 and 86/609/CEE) and approved by the Bioethics Committee of the University of Salamanca (reg.0000305). Six-week-old CD1 mice (Chen et al., 2017; Katare et al., 2016; Katare et al., 2013) were purchased from Charles River Laboratories (Barcelona, Spain). For at least one week before their experimental use, the animals were fed with a standard diet and *ad libitum* access to tap water without sterile conditions.

All animals were anesthetized through inhalation with a mixture of oxygen (0.8 L/min) and isoflurane (1.5–2 vol%), and an intraperitoneal injection of 0.1 mg/kg buprenorphine was administered to ensure an analgesic period of 8–12 h.

After hair removal, hindlimb ischemia was induced through ligation of the right femoral artery. The right femoral artery was excised from its proximal origin as a branch of the external iliac artery to the distal point where it bifurcates into the saphenous and popliteal arteries. The femoral artery was separated from the nerve so as to be occluded with a non-absorbable suture 6/0 Ethicon and cut using microsurgery scissors (Fine Science Tools). Finally, the skin incision was closed with an absorbable suture 5/0 Ethicon and wiped with betadine. The contralateral hindlimb was used as the internal control.

### Cell and Hydrogel Administration

After surgery, the following experimental groups were established: 1) mice with the ELR hydrogel (30 μL) (*n* = 11), 2) mice with 0.5 × 10^6^ BM-MSCs (30 μL) (*n* = 11), 3) mice with the ELR hydrogel +0.5 × 10^6^ BM-MSCs (30 μL) (*n* = 12), and 4) control mice (*n* = 8); only RPMI was administered.

For the ELR hydrogels containing BM-MSCs, 0.5 ×·10^6^ cells were added to REDV-N_3_ and this mixture was subsequently mixed with the same volume of HRGD6-Cyclo for a final volume of 30 µL/hydrogel. The cell suspension in the ELR mixture was immediately loaded into a syringe and injected into the right quadriceps muscle to achieve hydrogel formation. Non-combined ELR hydrogels (formed as aforementioned) and cells were injected similarly. Injections were administered 24 h after surgery.

In addition, a mouse without artery ligation was injected with both the ELR hydrogel and 0.5·10^6^ BM-MSCs (30 μL) and killed the day after for analysis of the cells inside the hydrogels.

### Monitoring and Quantification of Blood Flow

Tissue perfusion was monitored on the hindlimbs at days 0, 1, 7, 14, and 21 post surgical intervention. Laser Doppler perfusion imaging (LDPI) (Moor Instruments) was used to measure perfusion of both right and left limbs. LDPI allows the blood flow to be registered semi-quantitatively due to the movement of red cells that produces a frequency of change of the reflected light (Doppler effect). After limb hair removal and heating at 37° C with a thermal blanket to minimize temperature variations, different measurements were performed with mice placed in the dorsal position on foot regions. Analysis was completed by calculating the average perfusion of the different values obtained from right hindlimbs. As a basal blood perfusion, measurements were taken one day before performing the ligation of the femoral artery. Percentage of blood flow at days 1, 7, 14, and 21 post surgical intervention vs. basal blood flow was calculated. Measurements were carried out in eight mice of the control group, 11 mice of the hydrogel group, 11 mice of the MSC group, and 12 mice of the MSC + ELR group.

### Tissue Preparation

The mice were killed 21 days after surgery, and both paws were removed. Both right and left hindlimbs were fixed with 4% formaldehyde at room temperature for about 48 h. Bones were carefully removed by cutting along the muscle tissue so that the whole paw could be analyzed on different sections. Quadriceps tissues, located where MSCs and ELR were injected, were processed to be embedded in paraffin and serially cut at 3-μm intervals (Jeong et al., 2020; Yin et al., 2019).

### Three-Dimensional (3D) Culture

BM-MSCs (5·10^5^ cells/hydrogel) were mixed with the REDV-N_3_ and this mixture was added to the HRDG6-Cyclo solution (1:1 volume ratio) for a total volume of 50 µL per hydrogel. This volume ensured 1.5 mm of thickness. Then, each hydrogel was pipetted into a well of a 96-well plate and incubated at 4°C for 10 min for homogeneous crosslinking of the ELR molecules. Subsequently, the plate was incubated at 37°C for 10 min with 100 µL of RPMI medium, which was changed every two days. The hydrogels together with the BM-MSCs were removed from the wells at days +1 and +21 and fixed with 4% formaldehyde in order to be embedded in paraffin and serially cut into 3-μm sections.

### Histological Analysis by Hematoxylin and Eosin Staining

Both three-dimensional culture samples and mice tissue samples were stained with hematoxylin and eosin (H&E) for general evaluation, according to standard protocols.

### CD45 Immunostaining in Muscles

Immune/inflammatory cell detection was also evaluated by CD45 immunostaining in quadriceps tissues. For this purpose, epitope unmasking was first performed using a citrate buffer at 100°C. The samples were then incubated with a primary anti–CD45 antibody (Abcam, ab10558) and subsequently with the secondary antibody OmniMap (Roche). Finally, samples were developed with DAB and counterstained with H&E.

### Histological Analysis by Masson’s Trichrome Staining

In order to analyze collagen distribution in quadriceps tissues, a Masson Trichrome Goldner Kit (Bio Optica, 04–011802) was used according to the manufacturer’s instructions.

### Identification of BM-MSCs and the ELR-Based Hydrogel

BM-MSCs were identified by immunofluorescence staining. The samples were incubated in sodium citrate 10 mM and 0.05% Tween-20, pH 6 at 100 °C for 20 min, rinsed with cold PBS, blocked with 0.2% v/v Triton X-100 and 5% v/v donkey serum in PBS for 1 h at room temperature, and incubated with mouse anti-human mitochondria (dilution 1:1,000, Millipore) overnight at 4°C. Finally, the slides were incubated for 1.5 h in the dark with anti-mouse Alexa555 secondary antibody (dilution 1:1,000, Life Technology). Cell nuclei were stained with DAPI (Invitrogen). Images were obtained using a Leica SP5 confocal microscope.

Elastic fibers of the ELR-based hydrogel were detected with an elastic stain (HT25A-1KT, Sigma) according to the manufacturer’s instructions.

### Assessment of Capillary Density in Muscle Tissue

To analyze the capillary density in treated and untreated ischemic tissues, immunostaining of muscle slices was performed at day 21 post treatment. For that, samples were treated with Cell Conditioning 1 buffer (CC1) TRIS pH 8 during 32 min before incubation with the primary antibody. Subsequently, the primary anti–CD31 monoclonal antibody (ab182981, Abcam) was added at 1:200 dilution and incubated for 40 min at room temperature. OmniMap Discovery anti-Rb HRP (Ventana Medical Systems, Roche) was used as secondary antibody. Finally, the signal was detected using the Discovery ChromoMap DAB kit (Ventana Medical Systems, Roche). Vascularization was determined by the density of CD31 positively stained vessels, quantified as the number of capillaries per muscle fiber in ten fields per muscle. Four mice were analyzed for each condition.

### Statistical Analysis

Statistical analysis was performed with GraphPad Prism 7.0 software. The data are shown as the mean ± standard error of the mean (SEM). The Mann–Whitney test was applied to compare the *in vivo* data, and differences were considered statistically significant when *p*-values were <0.05.

## Results

### ELR-Based Hydrogel and BM-MSCs Promote Reperfusion in Ischemic Hindlimb

**FIGURE 1:**
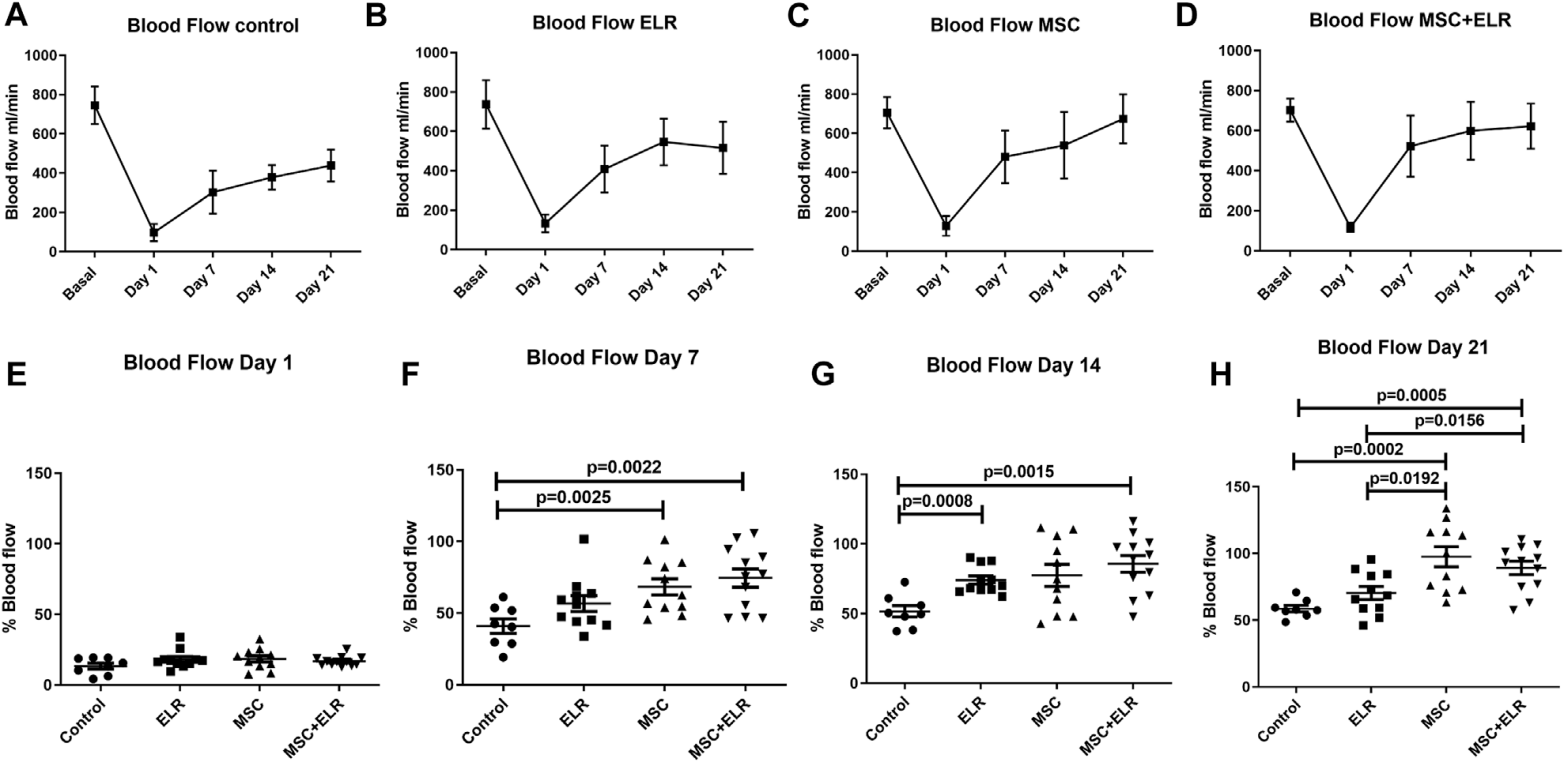
Evolution of tissue reperfusion after femoral artery ligation in CD1 mice. Representative lineal graphs of blood flow of **(A)** control (*n* = 8), **(B)** ELR biomaterial (*n* = 11), **(C)** MSC (n = 11), and **(D)** MSC + ELR (*n* = 12) groups. Percentage of perfusion vs. basal perfusion at days 1 **(E)**, 7 **(F),** 14 **(G),** and 21 **(H)** post surgical intervention vs. basal blood flow are represented in each experimental group. Data represent the mean ± SEM of four independent experiments. ELR: elastin-like recombinamer; MSCs: mesenchymal stromal cells.

When blood flow was evaluated in the ischemic right hindlimbs of control mice and mice injected with ELR-based biomaterial, BM-MSCs and BM-MSC + ELR biomaterial, we observed that it is progressively recovered in all experimental groups, including the control (Figures 1A–D). Reperfusion, measured as the percentage of blood flow vs. basal, was almost complete in the MSC [99.92% (72.82–116.2%)] and MSC + ELR [93.74% (75.59–103.8%)] groups at day 21. We detected a significant improvement of tissue reperfusion in MSC-only and MSC + ELR groups compared to the control group (*p* = 0.0025 and *p* = 0.0022, respectively) at day 7 post surgery (Figure 1F). At day 14, differences were significant in ELR-only and MSC + ELR groups versus the control group (*p* = 0.0008 and *p* = 0.0015, respectively) (Figure 1G). Finally, at day 21 post surgical intervention, we again found a significant improvement of tissue perfusion in both MSC and MSC + ELR groups in comparison with the control group (*p* = 0.0002 and *p* = 0.0005, respectively). We also found differences at this time point between ELR-only and MSC + ELR groups (*p* = 0.0156) and ELR-only and MSC-only groups (*p* = 0.0192) (Figure 1H). Representative images of LDPI images can be seen in Supplementary Figure S1.

### Histological Impact of MSC and ELR Administration

**FIGURE 2:**
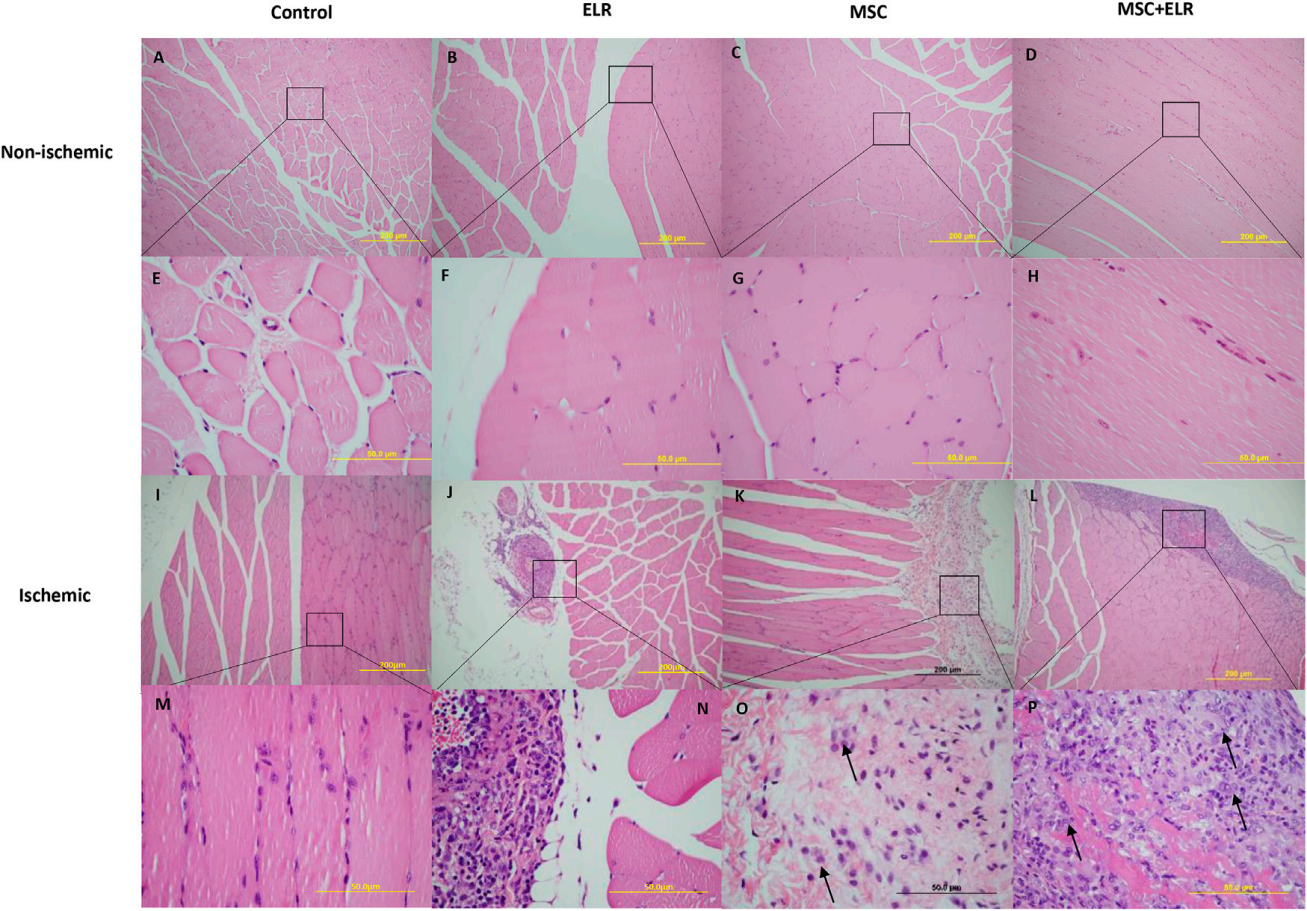
Morphology of muscle tissue sections. Representative images of ischemic and non-ischemic hindlimbs stained with H&E after 21 days post surgery are represented. **(A–D)** and **(I–L)** 10x amplification. **(E–H)** and **(M–P)** 60x amplification. ELR: elastin-like recombinamer; MSCs: mesenchymal stromal cells. Arrows point out MSCs.

**FIGURE 3:**
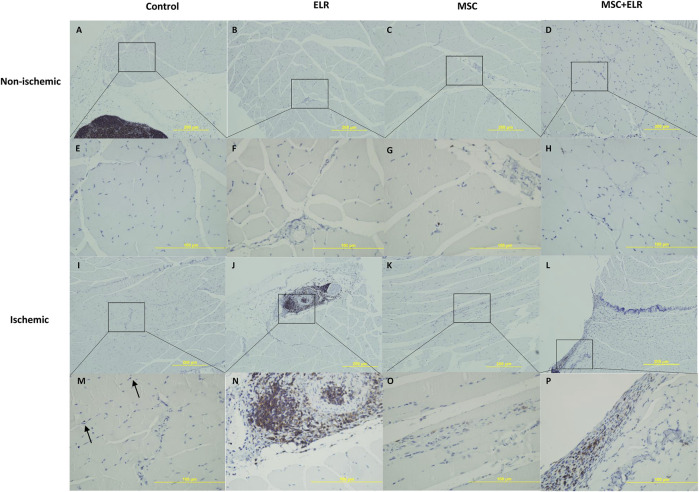
Immunohistochemical staining for CD45 to detect immune cells. Representative images of ischemic and non-ischemic hindlimbs stained for CD45 detection after 21 days post surgery are represented. Positive staining is indicated by the dark brown areas. **(A–D)** and **(I–L)** 10X amplification. **(E–H)** and **(M–P)** 40X amplification. ELR: elastin-like recombinamer; MSC: mesenchymal stromal cells. Arrows point out CD45 positive cells.

**FIGURE 4:**
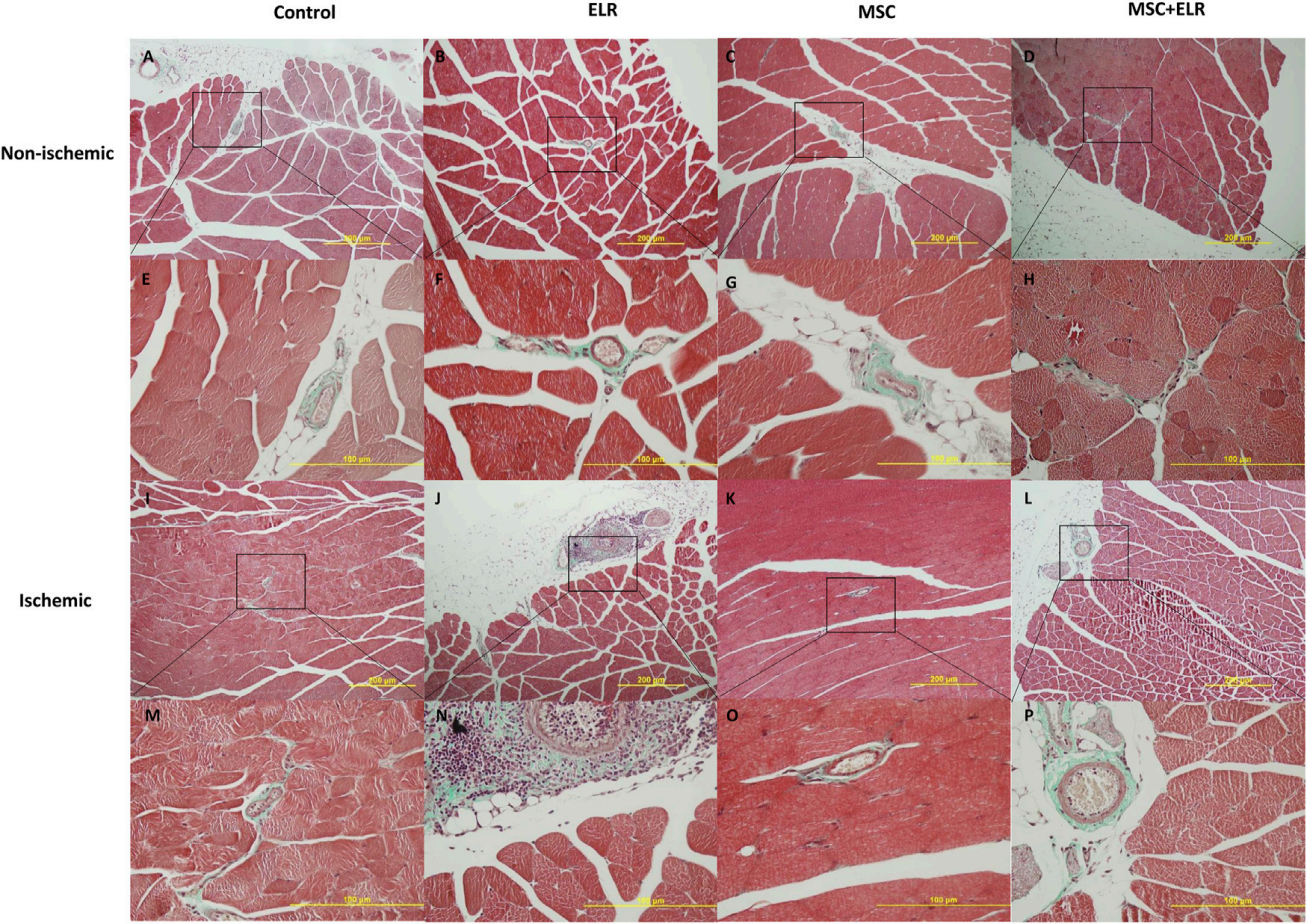
Masson’s trichrome staining for the analysis of collagen distribution. Representative images of ischemic and non-ischemic hindlimbs stained with Masson’s trichrome after 21 days post surgery are represented. **(A–D)** and **(I–L)** 10X amplification. **(E–H)** and **(M–P)** 40X amplification. ELR: elastin-like recombinamer; MSC: mesenchymal stromal cells.

At day 21 post injection, H&E staining for ischemic and non-ischemic muscles showed no major changes at the histological level between control and treated groups. The structural integrity of the muscle was almost perfectly preserved (Figure 2). In ischemic control muscles, we only detected few small inflammation sites and nuclear activation of muscle cells in some samples (in two of six samples) (Figures 2I,M) (Figures 3I,M). In quadriceps treated with the hydrogel, we could see some inflammatory reactions, granulomas, and augment of the fibroblast in some samples and a perivascular inflammation with the presence of acute inflammatory cells around the vessel in one sample (Figures 2J,N) (Figures 3J,N). This vessel may be a neovascularized one. In the case of muscles treated with BM-MSCs, they virtually did not present inflammatory reaction, but small collagen reaction in one sample was observed (Figures 2K,O). We observed some MSCs in the connective tissue both when they were injected alone (Figure 2O) or with the ELR-based hydrogel (Figure 2P). In muscles with MSC + ELR, we only identified some inflammatory cells with metachromatic cytoplasm and small nucleus (Figures 2L,P) (Figures 3L,P). In the same way, Masson’s trichrome staining showed the absence of muscular fibrosis. Collagen fibers were only present around vessels both in non-ischemic samples (left quadriceps) and in all groups, treated or untreated, of ischemic samples (right quadriceps), with no differences in the quantity or location of the collagen fibers among them (Figure 4). These results agree with those of previous studies where ELRs are shown to enhance skeletal muscle healing by inducing pro-regenerative type-2 macrophage polarization and a reduced deposition of collagen, which resulted in reduced fibrosis and an adequate environment for new muscle tissue development (Ibáñez-Fonseca et al., 2020).

### Identification of BM-MSCs and ELR-Based Hydrogel in Muscle Tissue

Given that the ELR-based hydrogel is biodegradable, we next assessed if BM-MSCs and the hydrogel persisted after 21 days both after *in vitro* culture and in the muscle tissue *in vivo*. To identify them *in vitro*, BM-MSCs with ELR biomaterial were cultured during 1 and 21 days in 3D. For *in vivo* identification, besides assessing mice killed at day 21, we used a mouse treated with MSC + ELR that was killed the following day.

**FIGURE 5:**
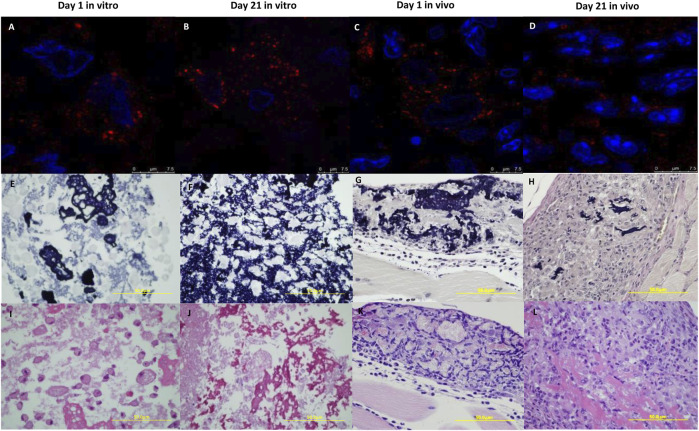
Identification of BM-MSCs and the ELR-based hydrogel *in vitro* and *in vivo*. ELR-hydrogels with BM-MSCs obtained from *in vitro* and *in vivo* sections were stained with human mitochondria antibody to identify BM-MSCs (red). Nuclei were stained with DAPI **(A–D)**. Elastic stain was used to detect the hydrogel (violet) **(E–H)**. In H&E sections, the ELR hydrogel was stained pink and BM-MSC nucleus was stained violet **(I–L)**. Experiments were conducted at 1 day **(A,E,I)** and 21 days **(B,F,J)** *in vitro* and at 1 day **(C,G,K)** and 21 days **(D,H,L)** *in vivo*. E-L 60x amplification.

Immunofluorescence staining with human mitochondria antibody showed the detection of MSCs in all the slides analyzed, both *in vivo* and *in vitro* at day 1 and day 21 (Figures 5A–D). Immunohistochemical staining for elastic fibers confirmed the identification of the ELR-based hydrogel both *in vivo* and *in vitro* at all timepoints, with noticeable reduction of hydrogel amount *in vivo* after 21 days due to biodegradation (Figures 5E–H). With hematoxylin and eosin (H&E) staining, we could also identify both structures in all experimental conditions (Figures 5I–L).

### ELR-Based Hydrogel and BM-MSCs Promote Angiogenesis in Ischemic Hindlimb

**FIGURE 6:**
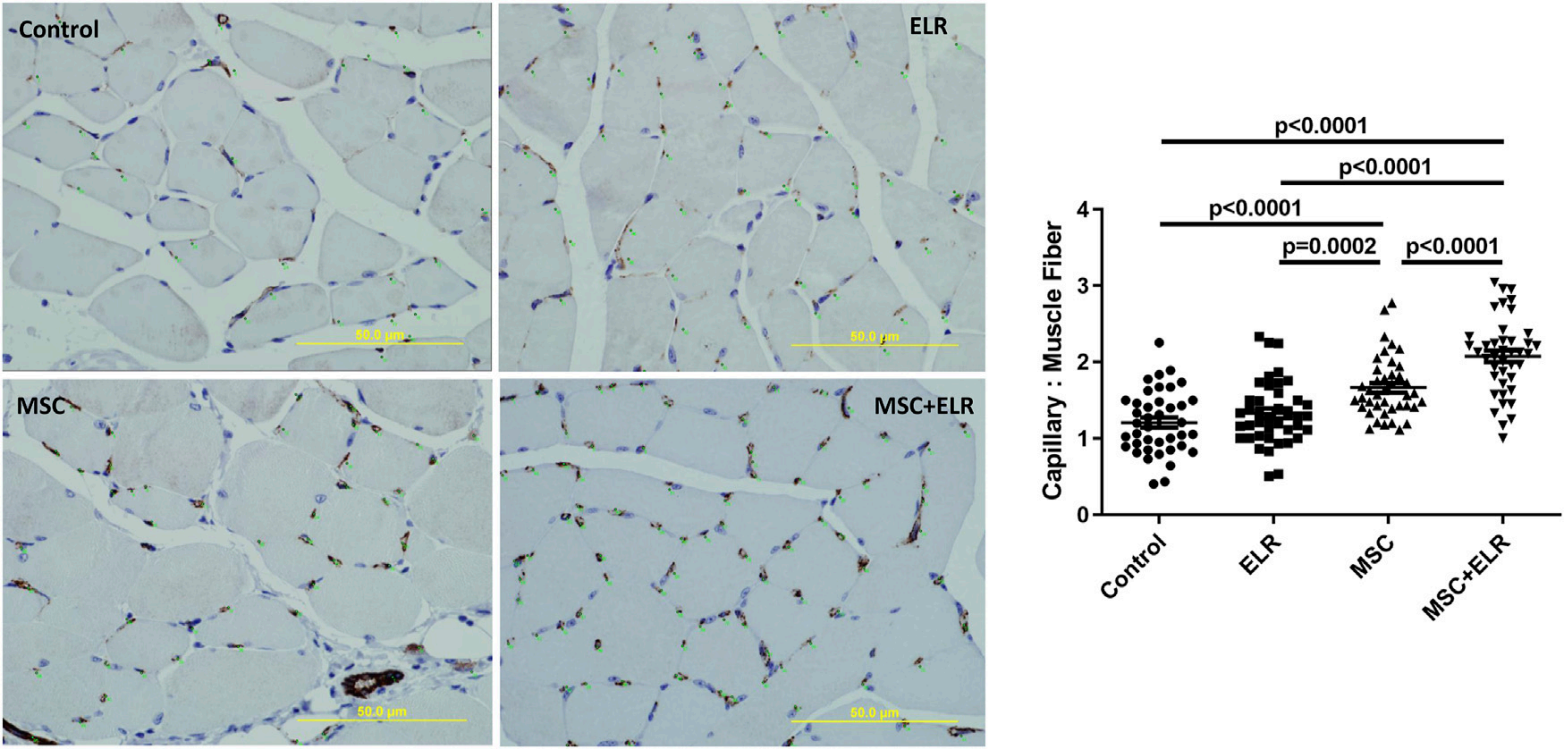
Vessel quantification in ischemic muscles 21 days after treatment with the ELR biomaterial, MSCs, or both in CD1 mice. Vessel density was quantified by immunostaining of ischemic muscles using CD31 antibody. Vascularization was determined by the density of CD31 positively stained vessels, quantified as the number of capillaries per muscle fiber. CD31 positive cells could be seen in brown due to DAB precipitation. Ten fields per muscle were analyzed. Quantification was carried out in four mice per condition. ELR: elastin-like recombinamer; MSCs: mesenchymal stromal cells.

Immunohistochemistry analysis was performed at day 21 post treatment to evaluate blood vessel density in treated and untreated ischemic muscles. We observed a significant increase in the number of capillaries per muscle fiber in mice treated with MSC-only or MSC + ELR in comparison with untreated mice (*p* < 0.0001) or mice treated only with the material (*p* = 0.0002, *p* < 0.0001, respectively). Moreover, the number of capillaries per muscular fiber was significantly higher in the MSC + ELR group than in the MSC group (*p* < 0.0001) (Figure 6).

## Discussion

In the current work, we demonstrate the ability of MSCs alone and in combination with an ELR-based hydrogel to promote neovascularization in an ischemic muscle. This is the first report in which the angiogenic potential of ELR-based hydrogels combined with human BM-MSCs has been assessed in an *in vivo* model of hindlimb ischemia.

With the development of regenerative medicine, stem cell transplantation has become an alternative potential treatment for ischemic diseases. Different types of stem cells have been tested in this context with different outcomes. It is known that BM is a reservoir of cells with angiogenic capacities. We have previously demonstrated that the administration of monocytes improves blood flow in ischemic tissues. However, CD133^+^ cells (including CD34^+^ cells and endothelial progenitor cells) produce a faster flow recovery (Sanchez-Guijo et al., 2010). It has been argued that the use of selected populations instead of total mononuclear cells (MNCs) or whole BM may exclude some important cell subsets with angiogenic properties. However, with the infusion of total MNCs, there is a risk of side effects due to the infusion of cells with proinflammatory activity (Higashi et al., 2004; Tateishi-Yuyama et al., 2002). In this regard, some reports have described that MSC transplantation induces a better improvement than MNC both in ischemic hindlimb or myocardial infarction (Iwase et al., 2005; Mazo et al., 2010; Qi et al., 2008). Iwase et al. described how the number of endothelial cells derived from transplanted cells was higher in rats infused with MSCs than in rats infused with MNC, and vascular smooth muscle cells derived from transplanted cells were detected only in that group of study. One of the facts that may contribute to these results is that MSCs are less sensible to starvation, hypoxia, or to an ischemic environment than MNCs (Ferro et al., 2019). Another advantage in the use of MSCs compared to MNCs is that the former are adherent and thus able to undergo *in vitro* expansion from a small BM sample. Furthermore, MSCs secrete a wide variety of angiogenic factors compared to other subsets of stem cells, including vascular endothelial growth factor (VEGF), stromal derived factor 1 (SDF-1), fibroblastic growth factor (FGF), or transforming growth factor (TGFβ) (Al-Khaldi et al., 2003; Kinnaird et al., 2004; Premer et al., 2019), and have shown to form capillary-like structures *in vitro* under determinate conditions (Annabi et al., 2003). These attributes, together with their immunomodulatory properties and their regenerative capacity, make them excellent potential candidates for the treatment of hindlimb ischemia. MSCs have been tested in several preclinical studies (Iwase et al., 2005; Al-Khaldi et al., 2003; Kinnaird et al., 2004), and although the majority of the reported clinical trials used autologous MNC, there are also some studies where MSCs have been and are currently being assessed for this purpose in patients, either delivered locally through the intramuscular route or systemically, with promising results (Soria-Juan et al., 2019; Soria-Juan et al., 2021; Gupta et al., 2013). MSC effects may depend more on secreted soluble factors than on engraftment and transdifferentiation. Sometimes, a great improvement in perfusion is reported despite the lack of detectable engrafted cells (Kinnaird et al., 2004). In fact, it has been described that MSCs do not transdifferentiate *in vivo* into vascular or muscle cells but persist acting in a paracrine manner (Bortolotti et al., 2015) The release of substances into the environment through extracellular vesicles can also be an important component of the therapeutic action of MSCs (Preciado et al., 2019; Preciado et al., 2021). Recent studies have indicated that these EVs can promote angiogenesis and repair injury in ischemic tissues (Arslan et al., 2013; Xin et al., 2013), and the transference of miRNAs from these EVs to endothelial cells promotes vessel formation (Gong et al., 2017). In fact, it has been shown that only the infusion of EV, isolated from the MSC supernatant, attenuates limb ischemia and promotes angiogenesis in a murine model (Hu et al., 2015).

Biomaterial scaffolds can be used as a substrate providing not only mechanical support but also essential signaling molecules for angiogenesis, differentiation, and proliferation of cells. These scaffolds can be produced from different biomaterials with different composition and properties. Currently, chemical biodegradable hydrogel scaffolds hold promising results for improving angiogenesis both *in vitro* and *in vivo*. However, in most cases, scaffolds alone may not be sufficient to significantly improve the vasculature of the ischemic tissues (Serbo and Gerecht, 2013). Some researchers have used biomaterials as an anchor to attach bioactive molecules as VEGF that enhances angiogenesis (Shivani Singh et al., 2011; Flora et al., 2019; Flora et al., 2019), but the most promising results have been obtained using biomaterials as scaffolds for supporting cell growth and differentiation (Staubli et al., 2017; Cipriani et al., 2019; Amani et al., 2021). A significant challenge in tissue engineering is poor engraftment or non-uniform proliferation of cells in the scaffold. Biocompatibility, cell-friendly behavior, and mechanical properties such as thermoresponsiveness, velocity of degradation, gelation, or porosity of the material are key factors for rapid formation of blood vessels that is critical to avoid cell death (Mehdizadeh et al., 2015). There should also be a balance between degradation and preservation of scaffold integrity during vessel formation process until enough tissue has been formed. Different biomaterials have been used for cell delivery in regenerative medicine applications, including natural and synthetic ones (Wang et al., 2019). However, both types present disadvantages that have not been overcome so far, such as batch-to-batch variability and uncontrolled degradation for natural ones or lack of biocompatibility and bioactivity for synthetic ones, which have hampered their development and application (Facklam et al., 2020). On the other hand, ELR biomaterials combine several advantages, from their recombinant origin to include bioactive domains and accurate production, to a protein nature inspired in elastin that makes them highly biocompatible (Ibáñez-Fonseca et al., 2019). While they have not been strictly compared to other biomaterials, they have already shown to promote MSC engraftment in an osteochondral regeneration model (Valencia et al., 2016). Moreover, the composition of our ELR-based hydrogel permits immediate gelation by click-chemistry upon administration into an organism, conferring the benefit of being an injectable scaffold for a minimally invasive administration.

Regarding blood flow, we can see in our experiments, as it has been previously described, that it can be partially recovered after iliac artery ligation even in the control group (Iwase et al., 2005; Sanchez-Guijo et al., 2010). However, we can observe that the infusion of MSCs significantly improves tissue reperfusion. Several works support these results with MSCs alone (Al-Khaldi et al., 2003; Kinnaird et al., 2004; Iwase et al., 2005; Gupta et al., 2013). In accordance with these data, Lian et al. described that MSCs derived from iPSCs attenuate limb ischemia (Lian et al., 2010) and also their EV (Hu et al., 2015). In addition, the administration of MSCs combined with the ELR-based hydrogel does not increase the blood flow significantly when compared to mice treated with MSCs alone in any of the three time points analyzed. In this regard, although published information is scarce, there is one report describing different results with an increase in the reperfusion ratio with the administration of a Nap-GFFYK-Thiol hydrogel together with MSCs when compared to MSCs alone at day 28, and this difference may be attributed to the different nature of the material (Huang et al., 2018). Upon macroscopic and histological evaluation, we did not observe major changes despite minor inflammation sites in muscles where the biomaterial was injected alone or with MSCs and a small collagen reaction in muscles injected with MSCs. In accordance with these findings, some groups have also described the absence of important inflammation response when ELR-based hydrogels are injected subcutaneously in mice (Ibáñez-Fonseca et al., 2018; Ibáñez-Fonseca et al., 2020; González-Pérez et al., 2021). It has also been reported that there is no effect on collagen fiber distribution neither in a situation of ischemia nor after treatment with biomaterials or MSC (Amani et al., 2021) as is observed in our samples. However, in other studies, it is shown how fibrosis can be increased in ischemic hindlimbs and collagen deposition can be reduced after through MSC and biomaterial treatment (Huang et al., 2018; Marsico et al., 2021). These findings suggest that ELR-based hydrogels are biocompatible. Moreover, it has also been described that ELR-based hydrogels could even modulate macrophage polarization toward an anti-inflammatory phenotype in ischemic-induced inflammation environments (Marsico et al., 2021).

We have demonstrated that MSCs persist together with the biomaterial both *in vivo* and *in vitro* 21 days after culture or injection. This is in accordance with a previous work where we have observed proliferation of HUVEC cells within the material for at least 9 days and also that human MSCs were viable for at least 4 weeks in terms of bioluminescence emission when embedded in ELR-based hydrogels and injected subcutaneously into mice (Ibáñez-Fonseca et al., 2018). We have also observed in a previous work how the viability curve trend of MSCs embedded in ELR for 15 days was similar to the typical cell-growth behavior of MSC. In addition, these MSCs showed even more elongated and flat morphology (Cipriani et al., 2019). Other works describe an increased number of protrusions in MSC, demonstrating their adhesion to the biomaterial *in vitro* and how the hydrogel was present after 28 days *in vivo* (Staubli et al., 2017; Coletta et al., 2017). It seems that the REVD tetrapeptide has a key role in this adhesion (Girotti et al., 2020). This persistence of MSCs after 21 days is not usual when injected alone (Kinnaird et al., 2004). It has been demonstrated that biomaterials enhance MSC retention and also their secretome preservation (Brennan et al., 2020). Finally, by specific immunostaining for CD31 we observed a higher blood vessel density when mice were treated with MSCs than control, but the effect was higher when mice were treated with MSCs and the biomaterial. We did not observe significant differences with the administration of biomaterial alone. Similar results were obtained in a work where constructs of ELR-based hydrogels alone or with stromal vascular cells were inserted in subcutaneous pockets in rats. Using CD31 staining, they also showed that the presence of cells significantly enhanced the vessel ingrowth into the implants compared to the cell-free hydrogels (Staubli et al., 2017). In contrast to our results, it has also been proven that angiogenesis and proliferation of endothelial cells can be enriched only with the administration of ELR-based hydrogels (Marsico et al., 2021) or even other types of hydrogels (Huang et al., 2018). These discrepancies could be associated to different modifications in the ELR-based hydrogel.

We can conclude that the addition of an ELR-based hydrogel improves the therapeutic effects of MSCs on revascularization of ischemic tissues. This effect could be associated to many mechanisms. It has been demonstrated that the use of scaffolds could improve the viability and engraftment of the implanted cells at injury sites, and it can also change their paracrine effects and capability of interaction with the host tissue. Furthermore, the retention of different molecules released by MSCs in the scaffold could also help in enhanced angiogenesis (Shivani Singh et al., 2011) (Brennan et al., 2020) (Ciuffreda et al., 2018) (Huang et al., 2018).

## Data Availability

The raw data supporting the conclusion of this article will be made available by the authors, without undue reservation.
