# Supplementary figures and images for: Mesenchymal Stromal Cells Combined With Elastin-Like Recombinamers Increase Angiogenesis In Vivo After Hindlimb Ischemia
Source: Front Bioeng Biotechnol. 2022 Jun 23;10:918602. doi: 10.3389/fbioe.2022.918602 (PMC9260019; doi:10.3389/fbioe.2022.918602)

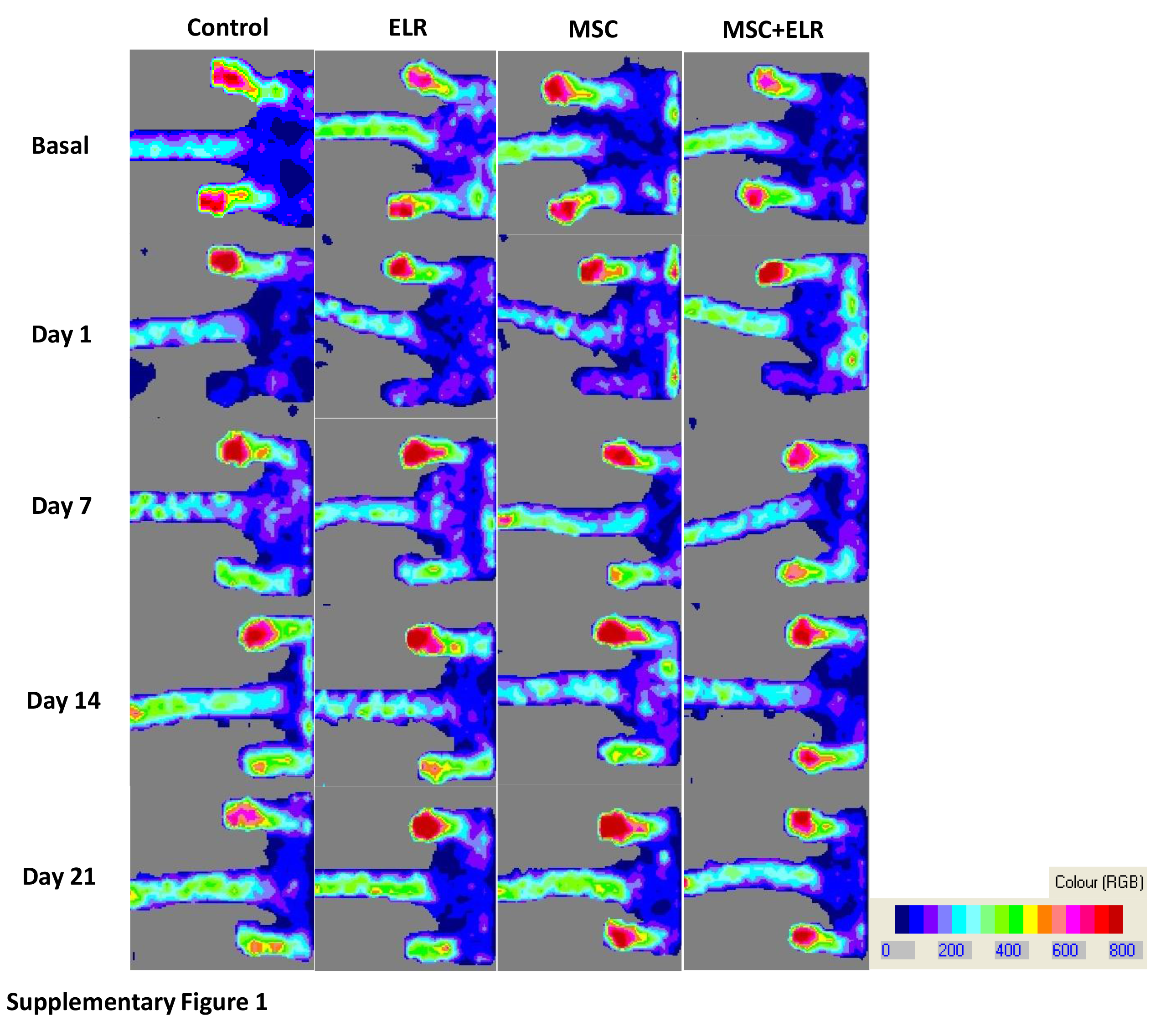

Supplement: Supplementary file 1 [file Image1.TIF]
